# Intermuscular Adipose Tissue as a Risk Factor for Mortality and Muscle Injury in Critically Ill Patients Affected by COVID-19

**DOI:** 10.3389/fphys.2021.651167

**Published:** 2021-05-06

**Authors:** Andrea P. Rossi, Leonardo Gottin, Katia Donadello, Vittorio Schweiger, Piero Brandimarte, Giulia A. Zamboni, Alessandro Florio, Riccardo Boetti, Gaia Pavan, Mauro Zamboni, Enrico Polati

**Affiliations:** ^1^Department of Medicine, Geriatrics Division, University of Verona, Verona, Italy; ^2^Department of Cardio-Thoracic Anaesthesia and Intensive Care, University of Verona, Verona, Italy; ^3^Department of Anaesthesia and Intensive Care B, University of Verona, Verona, Italy; ^4^Department of Public Health and Diagnostics, Radiology Division, University of Verona, Verona, Italy

**Keywords:** SARS-CoV-2, COVID-19, obesity, intermuscular adipose tissue, intensive care unit

## Abstract

**Background:**

Muscular fatigue and injury are frequently observed in critically ill COVID-19 patients. The aim of this study was to determine whether different muscle adipose tissue depots are associated with mortality and muscle damage in patients affected by COVID-19 admitted to the ICU.

**Methods:**

CT images were obtained in 153 ICU patients with COVID-19 (121 males and 32 females). Height, weight, body mass index (BMI), C-reactive protein, Creatine PhosphoKinase (CPK), muscle density, and intermuscular adipose tissue (IMAT) were measured.

**Results:**

Participants in the highest tertile of IMAT/muscle had the shorter 28-day survival from ICU admission as compared to subjects in the first tertile. Estimates derived from the Cox proportional hazard models, after adjustment for age, sex, and BMI, confirmed the results of the survival analysis (HR 3.94, 95% CI: 1.03–15.09). Participants in the lowest tertile of muscle density had the shorter survival at 28 days from ICU admission as compared to subjects in the highest tertile (HR 3.27, 95% CI: 1.18–4.61), but the relationship was no longer significant when age was included in the model. Subjects in the second muscle density tertile did not show an increased risk.

Participants in the highest tertile of IMAT/muscle and those in the lowest tertile of muscle density showed both significantly higher CPK adjusted for weight values as evaluated during the first 8 days of hospitalization.

**Conclusion:**

Our data seem to suggest that higher levels of IMAT/muscle and low muscle density are both associated with higher risk of ICU mortality and muscle injury as evaluated with CPK level.

## Introduction

Significant deficits in upper and lower limb strength have been observed in patients with COVID-19, resulting in slow functional recovery after the hospitalization period. At the muscular level, COVID-19 exaggerated inflammatory response can lead to the characteristic symptoms of the mild flu-like form of COVID-19, characterized by myalgia and muscular exhaustion, but it can also determine a picture of severe muscle mass and function loss, more markedly observed at the proximal muscular level.

COVID-19 associated myopathy with severe proximal and bulbar weakness is characterized by elevated levels of IL-6 and Creatine PhosphoKinase (CPK) and the histological features of COVID-19 are mostly anecdotic and only a few case reports have been recently published on this subject. In these patients, examined muscles show perivascular inflammatory infiltration with endomysial extension and regenerating fibers (Zhang et al., 2020).

Obesity is a recognized risk factor for infection, hospitalization, and worse clinical outcomes in subjects affected by COVID-19 (Hajifathalian et al., 2020; Simonnet et al., 2020).

Moreover, obesity is characterized by an increase of lipid fat deposition inside and between muscle fibers, the so-called intramuscular and intermuscular adipose tissue (IMAT), respectively. Intramuscular fat accumulation resulting in low muscle density is associated with aging, insulin resistance, and sedentary lifestyle, whilst IMAT has been observed in patients with excess total adiposity and metabolic syndrome. Both kinds of fat depots are metabolically active tissues, able to secrete inflammatory cytokines and contribute to muscle damage (Beasley et al., 2009; Zamboni et al., 2019).

The aim of the present study was to evaluate if muscle fat depots were associated with increased mortality and may play a different role on muscle injury, as evaluated with CPK plasma concentrations, in critically ill COVID-19 patients admitted to our ICUs.

## Materials and Methods

### Study Design and Participants

The main study population consisted of 338 COVID-19 patients from the REINSURE-ARDS registry (62.46 ± 11.81 years; with mean body mass index (BMI) 29.20 ± 3.49 kg/m^2^, 17 women), consecutively admitted to the ICU of the University Hospital Integrated Trust of Verona between March 8th, 2020 and January 31st, 2021. All subjects had microbiologic confirmation of COVID-19 diagnosis by sampling of oro/nasopharyngeal swab (World Health Organization, 2020).

This study was approved by the ethical board of the University of Verona (Prog1946CESC, Prot 72485, November 12, 2018). Patient identification remained anonymous and written informed consent was obtained for participants. The ICU admission criteria and treatment decisions, including the determination of the need for intubation and type of administered antibiotic and antiviral therapy, were not standardized and were made by the attending medical team.

The present analysis was performed in a subsample of 153 subjects for which thoracic CT images were available.

### Anthropometric Measures

Patients’ height and weight were recorded at the beginning of hospitalization as previously reported (Rossi et al., 2016). BMI was calculated as the ratio between weight and height squared (kg/m2).

### Biochemical Measures

Repeated venous blood samples for C-reactive protein (CRP) and creatine phosphokinase (CPK) levels were obtained after overnight fasting as reported elsewhere (Rossi et al., 2021).

For the measurement of CRP, a kit was used for the quantitative determination of CRP in serum (CRP ROCHE applied on ROCHE/HITACHI COBAS 702, Roche Diagnostics GmbH, Mannheim, Germany). Measurement uncertainty declared 5.8%. The coefficient of variation (CV) of annual CQI to 6 mg/L is 6%.

Creatine phosphokinase concentration was determined on a fully automated analyzer (ANALIZER ROCHE/HITACHI COBAS 702, Roche Diagnostics GmbH, Mannheim, Germany). A multicenter evaluation of the within-run precision of the Advia 2120 system showed CV always lower than 0.7% for CPK.

### CT Analysis

Two trained operators measured muscle density and IMAT in a single image at the L3–L4 level with a dedicated workstation using Sliceomatic software (version 4.2; Tomovision, Montreal) (Ross et al., 1992). Density value ranges between −30 and −190 Hounsfield units (HU) and between 0 and +100 HU were considered for adipose tissue and muscle, respectively (Goodpaster et al., 2000).

The IMAT measurements were then normalized for the psoas muscle area, obtaining the psoas fat to muscle area ratio as reported elsewhere (Ahmadi et al., 2015). The mean psoas muscle density in HU, related with intramuscular adipose tissue, and the IMAT area to muscle area ratio (IMAT/muscle) were also calculated.

### Study Outcomes

Our primary outcome was to assess if IMAT area to muscle area ratio and mean psoas muscle density are associated with mortality. Our secondary outcome was to assess if IMAT area to muscle area ratio and mean psoas muscle density can influence the trend of critically ill induced myopathy as evaluated with CPK.

### Statistical Analysis

For the demographic and clinical characteristics of the patients, differences between groups were assessed using the Chi-squared test and Fisher’s exact test for categorical variables and the Student’s *t*-test or Mann–Whitney U test for continuous variables. Differences in mortality rates across IMAT/muscle tertiles and muscle density tertiles were preliminarily evaluated fitting Kaplan–Meier survival curves. Cox proportional hazard models and logistic regression models were used to assess the risk of death. Hazard ratio (HR) and 95% confidence intervals (95% CI) were estimated to investigate the association of the three tertiles groups for both variables, IMAT/muscle area ratio and mean muscle density, with the risk of mortality. Three models were fitted for each outcome: unadjusted, age-, and gender-adjusted and adjusted for BMI. Students *t*-test for unpaired data was used to compare the trend of CPK between tertiles of IMAT/muscle and mean muscle density. A *p*-value of 0.05 or less was considered to be statistically significant. Data analysis was conducted using SPSS 22.0 software.

## Results

Table 1 shows the main characteristics of the study population (mean ± SD) at baseline, divided into subjects who survived and who died. 153 subjects, 121 men and 32 women (20.9%) were included in the study. Subjects included in the present analysis were not different in age, sex distribution, BMI; all studied subjects required invasive mechanical ventilation.

**TABLE 1:** Characteristics of the study population according to mortality status.

	Total (SD) (*n* = 153)	Min-max	Survivors (SD) (*n* = 126)	Min-max	Deaths (SD) (*n* = 27)	Min-max	*p*
Age (years)	64.19 (9.98)	29–85	63.32 (10.50)	29–85	68.26 (5.55)	55–87	0.019
Sex (M) *n* (%)	121 (79.1%)		100 (79.4%)		21 (77.8%)		0.800
Height (m)	1.72 (0.07)	1.40–1.96	1.72 (0.07)	1.40–1.96	1.70 (0.07)	1.50–1.86	0.8
Weight (Kg)	87.24 (15.64)	46–157	83.54 (14.71)	52–150	88.37 (19.58)	46–157	0.234
BMI (Kg/m^2^)	29.30 (4.58)	20.45–45.38	28.25 (4.43)	21.37–41.90	30.58 (5.29)	20.45–45.38	0.039
Psoas area (cm^2^)	16.66 (9.37)	2.47–55.07	16.46 (9.40)	2.47–55.07	17.59 (9.34)	4.56–34.93	0.573
Psoas IMAT (cm^2^)	4.36 (3.77)	0.47–22.33	3.68 (2.53)	0.47–13.98	7.52 (6.31)	0.98–22.33	<0.001
Psoas muscle density (HU)	37.79 (8.55)	14.70–67.91	38.38 (8.58)	14.70–67.91	35.05 (8.03)	19.42–49.42	0.066
CRP (mg/dL)	122.02 (79.64)	4–410	115.95 (79.71)	4–410	150.33 (75.01)	2–281	0.042
CPK adjusted per weight (U/L/kg)	3.34 (6.42)	0.16–51.19	2.67 (4.76)	0.16–45.39	6.41 (10.95)	0.18–51.19	0.006
Hypertension	88 (57.5%)		68 (54%)		20 (74.1%)		0.085
Hearth failure	4 (2.6%)		2 (1.6%)		2 (7.4%)		0.144
Ischemic cardiopathy	13 (8.5%)		11 (8.7%)		2 (7.4%)		1.000
Neurological disorder	10 (6.5%)		9 (7.1%)		1 (3.7%)		1.000
Type 2 diabetes	29 (19%)		24 (19%)		5 (18.5%)		1.000
Thyroid	17 (11.1%)		12 (9.5%)		5 (18.5%)		0.185
Dyslipidemia	25 (16.3%)		19 (15.1%)		6 (22.2%)		0.392
Immunodepression	11 (7.2%)		7 (5.6%)		4 (14.8%)		0.105
Smoke habit	25 (16.3%)		22 (17.5%)		3 (11.1%)		0.571
Chronic renal failure	14 (9.2%)		9 (7.1%)		5 (18.5%)		0.075
Cancer	15 (9.8%)		8 (6.3%)		7 (25.9%)		0.006

*SD, standard deviation; BMI, body mass index; IMAT, intermuscular adipose tissue; HU, Hounsfield units; CRP, C reactive protein; CPK, creatine phosphokinase.*

Survivors showed lower age, BMI, IMAT area, higher CRP, and CPK levels as compared with death subjects. CRP level was significantly higher in subjects in the highest IMAT/muscle tertile as compared with subjects in the lowest tertile, while subjects with low muscle density did not (data not shown in Table 1).

**FIGURE 1:**
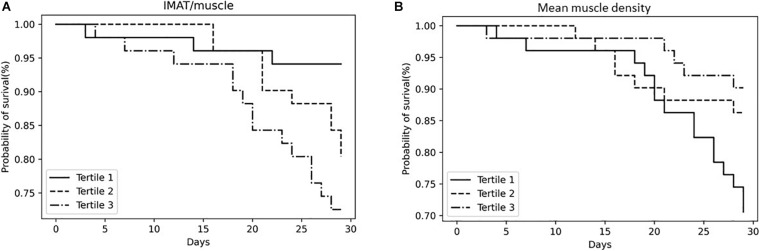
Kaplan–Meier survival curves for all-cause mortality according to IMAT/muscle tertiles **(A)** and mean muscle density tertiles **(B)**. IMAT, intermuscular adipose tissue.

Figure 1A shows that participants in the highest tertile of IMAT/muscle had a shorter survival at 28 days from ICU admission as compared to subjects in the first tertile. Estimates derived from the Cox proportional hazard models, unadjusted confirmed the results of the survival analysis (HR 5.22, 95% CI: 1.50–18.18). After adjustment for age and sex, this association was still significant (HR 3.91, 95% CI: 1.06–14.42), even when BMI was included in the model (HR 3.94, 95% CI: 1.03–15.09).

**FIGURE 2:**
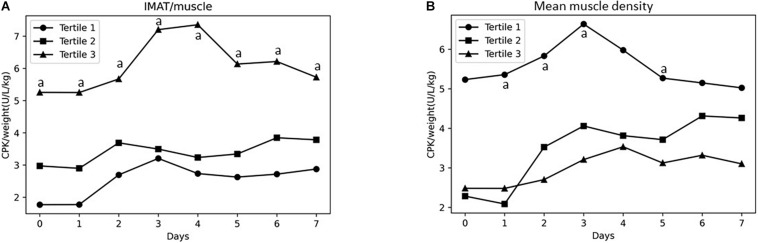
Changes during the first 7 days from admission in ICU in CPK adjusted for weight level across IMAT/muscle tertiles **(A)** and mean muscle density tertiles **(B)**. ^a^*p* < 0.05.

Survival of the study population stratified based on muscle density tertiles is shown in Figure 1B. Participants in the lowest tertile of muscle density had a shorter survival at 28 days from ICU admission as compared to subjects in the highest tertile. Estimates derived from the Cox proportional hazard models, unadjusted confirmed the results of the survival analysis (HR 3.27, 95% CI: 1.18–4.61), but the relationship was no longer significant when age was included in the model. Subjects in the second tertile of both, IMAT/muscle and muscle density, did not show an increased risk. Participants in the highest tertile of IMAT/muscle showed significantly higher CPK adjusted for weight values at baseline, day 1, 2, 3, 4, 5, 6, and 7, as compared to the first tertile (Figure 2A). Similarly, as shown in Figure 2B, subjects in the lowest muscle density tertile showed higher values of CPK adjusted values at baseline, day 1, 2, 3, and 5, as compared to the third tertile.

## Discussion

Our study shows that in COVID-19 intubated critically ill subjects with myosteatosis, as evaluated with CT, have higher ICU mortality risk. Moreover, we observed that both, subjects with higher IMAT/muscle ratio or low muscle density, experienced greater muscle injury, as evaluated with CPK levels, during the first 7 days of hospitalization in ICU.

This is in line with previous observations by Perkisas et al. (2018) who showed in a population of 302 geriatric patients that intramuscular adipose tissue, as evaluated with CT, was related to mortality during 4 years of follow-up.

Another prospective longitudinal study, including 1,652 African ancestry men, showed an independent association between myosteatosis and all-cause mortality during the 6 years follow-up (Zhao et al., 2016).

Our result that patients with higher IMAT are at higher risk of death than subjects with low muscle infiltration could be partially explained by the fact that IMAT is increased in obese subjects (Correa-de-Araujo et al., 2017). Indeed, the association remained significant after adjustment for age and sex, even when BMI was included in the model. On the contrary, after adjustment for age, the association between muscle density and mortality was no longer significant.

Subjects with higher IMAT/muscle showed higher CRP, while subjects with low muscle density did not.

Our data seems to suggest that the obese phenotype affected by severe COVID-19 with myosteatosis shows higher levels of inflammation and is at higher risk for unfavorable health outcomes.

COVID-19 is generally characterized by generalized weakness, myalgias, and elevated creatine kinase levels occurring in one-quarter of symptomatic patients (Guan et al., 2020; Guidon and Amato, 2020).

The pathophysiological mechanism associated with the elevation in creatine kinase level is still unclear.

Only a few reports characterized histologically muscle damage in COVID-19 subjects to investigate the relationship with CPK increase, but the oil-red O and perilipin staining were not quantified (Manzano et al., 2020; Zhang et al., 2020).

We observed an association between IMAT quantity and muscle damage, as evaluated with CPK level, in the first ICU days.

We can hypothesize that both high IMAT and low skeletal muscle density representation can be considered as a marker of muscle vulnerability.

In fact, ectopic fat deposition out of the subcutaneous depot is associated with elevated circulating proinflammatory cytokines (Beasley et al., 2009). Previous *in vitro* studies have demonstrated that adipokines have an effect at the level of the muscle cell (Takegahara et al., 2014) and that they can act on muscle at a local level via paracrine signaling (Bryniarski and Meyer, 2019). Intriguingly, both IMAT and pro-inflammatory adipokines, TNFα and IL-6, were found to be locally increased in the paretic leg of stroke survivors (Hafer-Macko et al., 2005), suggesting that the inflammatory milieu could be locally conditioned by IMAT quantity. Only systemic CRP levels were available for our patients and they were increased in subjects with higher IMAT quantity and a higher level of inflammation could partially explain the association between IMAT and muscle damage observed in our study.

Moreover, IMAT infiltration is correlated with greater skeletal muscle myostatin and P-CDK2 (Tyr15) in sedentary individuals, and its training-induced change is accompanied by reductions in myostatin and P-CDK2 (Tyr15), which promote adipogenesis and inhibit myogenesis (Konopka et al., 2018).

We observed that not only IMAT interspersed between muscle bundles, but also intramyocellular lipids could play a role in muscle damage in critically ill patients affected by COVID-19.

In fact, the accumulation of “toxic” lipids such as ceramides and diacylglycerols in muscles promotes not only metabolic consequences, such as insulin resistance but also skeletal muscle dysfunction with reduction of muscle fiber number, fiber size, contractility, mitochondrial dysfunction, and apoptosis. Intermuscular lipids are associated with higher levels of myostatin, IL-6, and macrophages infiltration, observed in subjects with obesity (Bodell et al., 1985; Hittel et al., 2009).

We can therefore hypothesize that different factors, related to both intermuscular and intramyocellular adipose tissue deposition, can interfere with satellite cell activation and myoblast proliferation and differentiation, which represent necessary steps for muscle repair processes after COVID-19 induced muscle injury. The subsequent muscle damage induced by the exaggerated inflammatory response observed in COVID-19 patients is associated with significant strength deficit both of the upper and the lower limbs, a phenomenon even more relevant in people with obesity, leading to worse clinical outcomes, as higher mortality and longer hospitalization.

Some limitations should be acknowledged. First, muscle density is related to, but not a precise measure of intramuscular fat content (Hausman et al., 2014). Secondly, inflammatory cytokines levels, such as IL-6 and TNF-alpha were not available for the whole study population, and further insight into the relationship between systemic inflammation and muscle local consequences was not possible. Lastly, this is a cross-sectional study and further longitudinal imaging and/or histological studies are needed to better clarify the impact of COVID-19 induced inflammation on muscle damage in hospitalized subjects and the possible role of different muscle fat depots.

## Conclusion

In conclusion, muscle quality measurements from thoracic imaging performed during the hospital stay of COVID-19 critically ill patients could provide valuable information on health outcomes, such as length of stay, in-hospital mortality, or post-COVID-19 functional recovery.

## Data Availability Statement

The raw data supporting the conclusions of this article will be made available by the authors, without undue reservation.

## Ethics Statement

The studies involving human participants were reviewed and approved by the Verona University Ethical Committee. The patients/participants provided their written informed consent to participate in this study.

## Author Contributions

LG, EP, and KD designed the research. EP, AF, KD, RB, and GP conducted the research. AR and LG had primary responsibility for final content and analyzed the data or performed the statistical analysis. AR, PB, and GZ collected and analyzed the CT images. AR, LG, KD, EP, and MZ wrote the manuscript. All authors contributed to the article and approved the submitted version.

## Conflict of Interest

The authors declare that the research was conducted in the absence of any commercial or financial relationships that could be construed as a potential conflict of interest.
